# Grey mould control by oxalate degradation using non-antifungal *Pseudomonas abietaniphila* strain ODB36

**DOI:** 10.1038/s41598-020-58609-z

**Published:** 2020-01-31

**Authors:** Yeyeong Lee, Okhee Choi, Byeongsam Kang, Juyoung Bae, Seunghoe Kim, Jinwoo Kim

**Affiliations:** 1 Department of Plant Medicine, Gyeongsang National University, Jinju, 52828 Republic of Korea; 2 Institute of Agriculture & Life Science, Gyeongsang National University, Jinju, 52828 Republic of Korea; 3 Division of Applied Life Science, Gyeongsang National University, Jinju, 52828 Republic of Korea

**Keywords:** Applied microbiology, Bacterial techniques and applications

## Abstract

Grey mould is an important necrotrophic fungal pathogen that causes huge economic losses in agriculture. Many types of bacteria are used for biological control of grey mould via competition for space or nutrients and/or the production of antifungal metabolites. Oxalate is a key component of virulent necrotic fungal pathogens. In this study, we isolated non-antifungal oxalate-degrading bacteria (ODB) from the surfaces of oxalate-rich spinach and strawberries to investigate their ability to control necrotic fungal pathogens such as grey mould. A total of 36 bacteria grown on oxalate minimal (OM) agar plates were tested for oxalate-degrading activity. Five isolates exhibiting the highest oxalate degradation activity were subjected to molecular identification using 16S rRNA gene sequencing. Two isolates exhibiting non-antifungal activity were subjected to disease suppression assays using *Arabidopsis*–*Botrytis* systems. The isolate *Pseudomonas abietaniphila* ODB36, which exhibited significant plant protective ability, was finally selected for further investigation. Based on whole-genome information, the *p*seudomonad *o*xalate *d*egrading (*podA*) gene, which encodes formyl-CoA transferase, was analysed. The *podA*^−^ mutant did not inhibit *Botrytis* infection and oxalate toxicity; the defects were recovered by *podA* complementation. Purified PodA–His converted oxalate to formate and eliminated oxalate toxicity. These results indicate that *P. abietaniphila* ODB36 and PodA enzyme are associated with various aspects of grey mould disease inhibitory effects.

## Introduction

*Botrytis cinerea*, which causes grey mould, is an important necrotrophic pathogen that causes huge economic losses in agriculture^1^. Grey mould is known to attack over 200 crop hosts including vegetables, fruits, flowers, and post-harvest crops^1^. Grey mould can cause soft rotting of all aerial plant parts and produce prolific gray conidiophores and conidia typical of the disease^1^. *B. cinerea* causes seed contamination in flax, sunflower, and lettuce^2^. In Australia, seed contamination by *B. cinerea* has caused total crop failure in chickpea^3^.

Although agricultural systems typically rely on chemical pesticides to inhibit grey mould, fungicides are avoided in sustainable agriculture due to their effects on the well-being of humans and the surrounding environment^4^. Fungicide overuse has led to the emergence of resistant fungal strains^5^. During the past few decades, these issues have led many researchers to develop biological control methods using antagonistic bacteria. The modes of antagonistic bacterial activity include competition, antibiosis, lytic enzyme production, interference with pathogen activity and growth, volatility, and host resistance induction. However, most biological controls evaluated under laboratory conditions have tended to fail in the field^6^. Sulfur dioxide fumigation is usually used to control postharvest grey mould; however, because it decreases berry quality by absorption in detached berries, safe, effective, and economical alternatives are needed^7^.

Many necrotrophic pathogens including grey mould are known to infect various host plants via the production of oxalate, which is toxic to cellular organisms including mammals and plants^8,9^. Oxalate acts as a strong chelator of cations; it greatly oxidizes organic compounds in necrotrophic pathogens^10^, helps to reduce pH, thereby facilitating plant infection by pectolytic enzymes, and impedes defense signaling pathways in the host^11^. Therefore, degrading oxalate at the onset of host pathogen interaction should block the infection process. Oxalate-degrading microorganisms are useful alternatives to chemicals or fungicides. Oxalate-quenching *Pseudomonas fluorescens* PfMDU2 was shown to inhibit the mycelial growth of *Rhizoctonia solani*, which causes rice sheath blight^12^. The oxalate-degrading bacterial strain *Cupriavidus campinensis* was reported to protect *Arabidopsis thaliana* and crop species against *B. cinerea*^13^.

In human, oxalates are consumed when eating foods high in oxalates such as spinach, strawberries, coffee, tea, and chocolate, or are produced by intestinal microorganisms from metabolic precursors. In the human intestine, oxalate can combine with calcium, magnesium, potassium, or sodium to form less-soluble salts, which can lead to pathophysiological disorders, including hyperoxaluria, urolithiasis, and kidney failure^14,15^. To overcome these disorders, studies on the use of oxalate-degrading enzymes of intestinal microorganisms such as *Oxalobacter formigenes* and *Lactobacillus acidophilus* have been conducted^15,16^.

The objective of this study was to isolate, characterize, and evaluate oxalate-degrading bacteria (ODB) for the control of grey mould. We performed protein overexpression of the *p*seudomonad *o*xalate *d*egrading enzyme (PodA) from non-antifungal *Pseudomonas abietaniphila*. Our aim was to provide a new approach for the control of grey mould via the application of oxalate-degrading genetic resources.

## Results

### ODB isolation and oxalate-degrading activity

**Figure 1:**
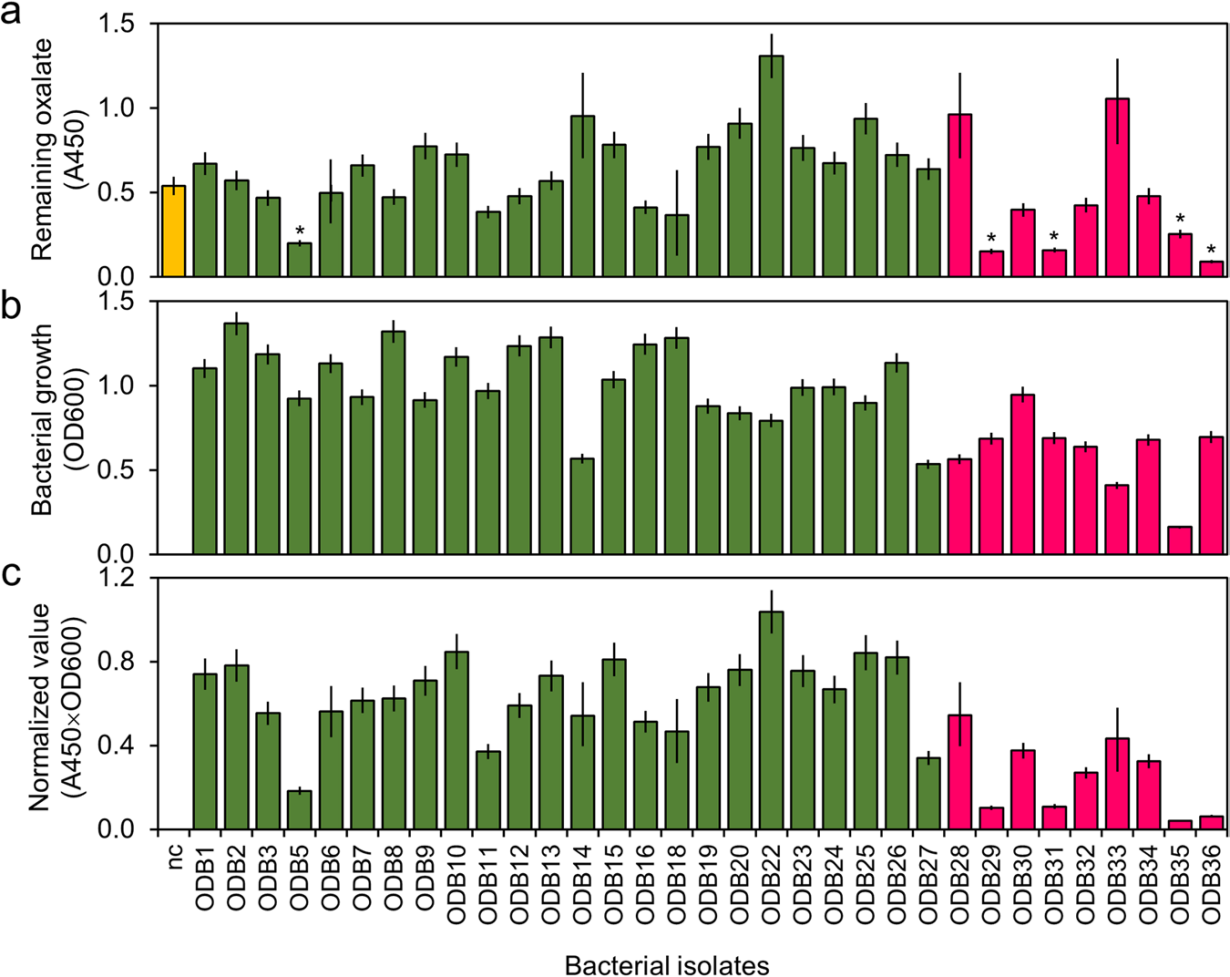
Oxalate degradation by bacterial isolates. (**a**) Absorbance measurement at A_450_. Oxalate in the supernatants was determined using an oxalate colorimetric assay kit. Bacterial isolates were grown in oxalate minimal (OM) medium supplemented with 1/10 TSB at 28 °C for 3 days. Cell-free supernatants were subjected to the sample preparation protocols described in Methods. The values report the amount of oxalate remaining in the OM medium. A total of 36 bacteria growing on OM agar plates were isolated from the surfaces of spinach (27 isolates; green) and strawberries (9 isolates; red). (**b**) Optical density (OD_600_) of the bacterial growth. (**c**) Normalised values calculated by multiplying the A_450_ value of the oxalate amount by the OD_600_ of the culture. The lower the value, the higher the degradation activity. Bacterial isolates ODB5, 29, 31, 35, and 36 exhibited significant oxalate degradation. Values are averages of triplicate assays and error bars represent the range. nc denotes the non-inoculated medium control. Asterisks denote significant differences from the control (*p < 0.05; Student’s *t* test).

A total of 36 bacteria grown on oxalate minimal (OM) agar plates (2 g Na_2_C_2_O_4_, 2.7 g K_2_HPO_4_, 0.9 g NaH_2_PO_4_, 0.9 g NH_4_Cl, 0.27 g MgSO_4_·7H_2_O, 0.009 g CaCl_2_·2H_2_O, and 0.0024 g FeSO_4_·7H_2_O in 1 L distilled water with 1.5% agar) were isolated from the surfaces of spinach (27 isolates) and strawberries (9 isolates). The oxalate-degrading activity of these 36 bacteria was tested in OM broth supplemented with 1/10 Bacto tryptic soy broth (TSB). Oxalate in the culture supernatants was determined using an oxalate colorimetric assay kit (Bio Vision Inc., San Francisco, CA, USA). Therefore, the lower the absorbance value at 450 nm (A_450_), the higher the degradation activity. When the non-inoculated media control (nc) supplemented with 15 mM sodium oxalate showed a value of 0.55, five isolates (ODB5, ODB29, ODB31, ODB35, and ODB36) exhibited significantly reduced values < 0.5 (Fig. 1a). Isolates with values higher than the control (nc) were suspected to have the ability to synthesize oxalate by themselves, and thus exhibited values > 0.55 (Fig. 1a). Each isolate showed a different growth pattern; in particular, the growth of ODB35 was relatively slow compared to other isolates (Fig. 1b). Because the growth of each isolate directly affects oxalate degradation, it is necessary to normalize values. Five isolates (ODB5, ODB29, ODB31, ODB35, and ODB36) had normalized values < 0.2, indicating that they had considerable oxalate-degrading activity (Fig. 1c). Three isolates (ODB4, ODB17, and ODB21) that did not grow in liquid culture were excluded.

### Bacterial identification and antifungal activity tests

To confirm the identities of the ODB, we amplified and sequenced the 16S rRNA genes of ODB5, ODB29, ODB31, ODB35, and ODB36, which exhibited significant oxalate-degrading activity. BLAST analysis of the 16S rRNA gene sequence of ODB5 showed 99% identity with *P. fluorescens*; those of ODB29, ODB31, and ODB36 showed 99% identity with *P. abietaniphila*; and that of ODB35 showed 99% identity with *Methylobacterium zatmanii*.

Isolates ODB5, ODB35, and ODB36 were selected for further antifungal activity testing. ODB5 exhibited antifungal activity against *B. cinerea*, *Alternaria alternata*, and *Saccharomyces cerevisiae*, whereas ODB35 and ODB36 showed no antifungal activity (see Supplementary Fig. S1).

### Disease suppression assay

**Figure 2:**
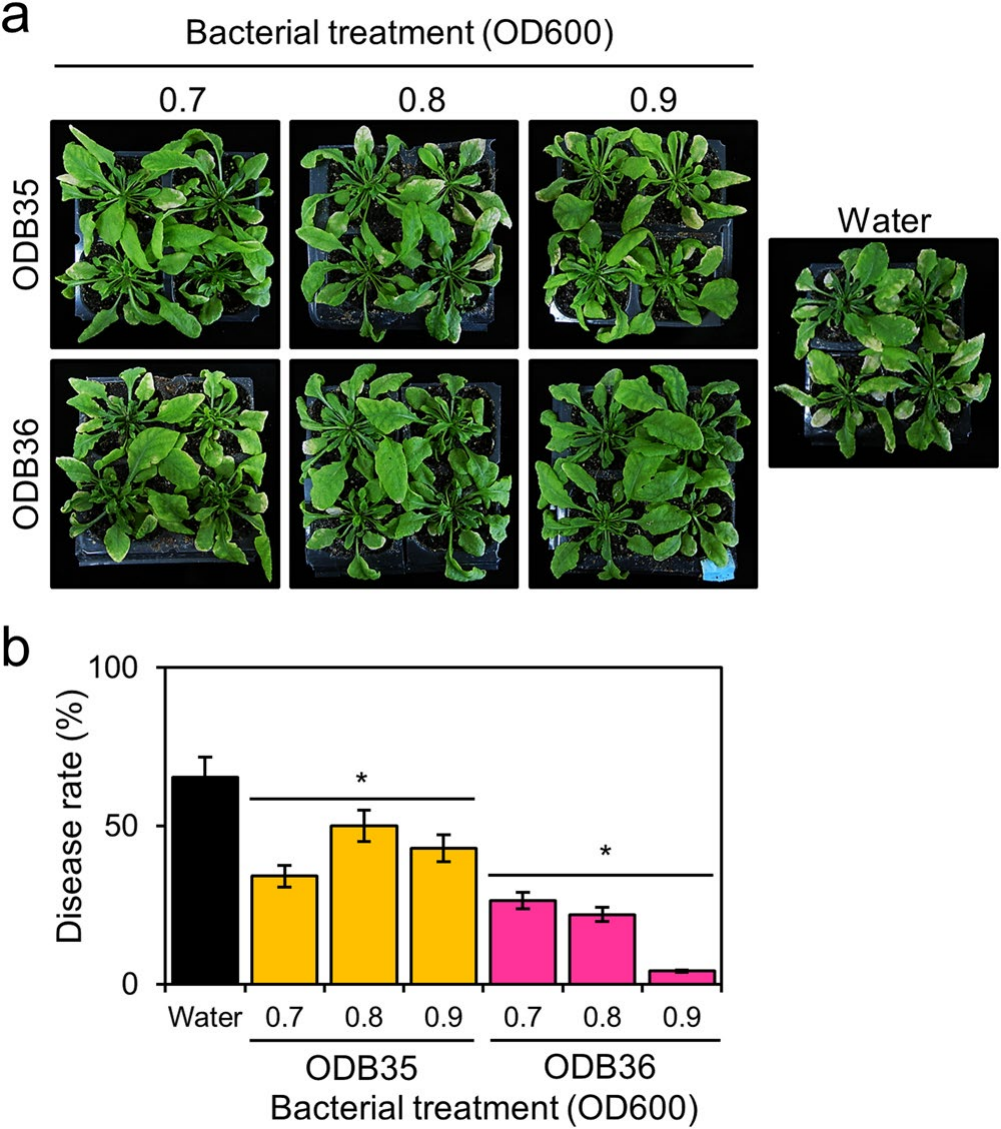
Grey mould disease suppression by ODB35 and ODB36. (**a**) Image captured 16 days after inoculation; (**b**) disease rate (%). Three-week-old *Arabidopsis* plants were sprayed with the ODB suspension. After incubation for 2 days, a *B. cinerea* spore suspension (5 × 10^5^ spores/mL) was prepared using sterilised water. The spore suspension was sprayed until run-off occurred. Inoculated plants were placed in an opaque plastic box lined with saturated paper towels, the lids were removed after 2 days, and disease development was observed for 16 days and measured. ODB36 exhibited significant disease suppression depending on treatment concentration. Values are averages of triplicate assays; error bars represent the range. Asterisks denote significant differences from the water control (*p < 0.05; Student’s *t* test).

To investigate disease suppression of ODB35 and ODB36, which exhibited no antifungal activity, we used the standard *A. thaliana*–*B. cinerea* (plant–pathogen) system. After 16 days of inoculation, water-sprayed plants exhibited a 65% disease rate against grey mould, whereas ODB36-sprayed plants exhibited significant disease suppression depending on the treatment concentration (Fig. 2). However, ODB35 exhibited no significant disease suppression compared with ODB36 (Fig. 2).

### Phylogenetic analysis and sequence alignment

**Figure 3:**
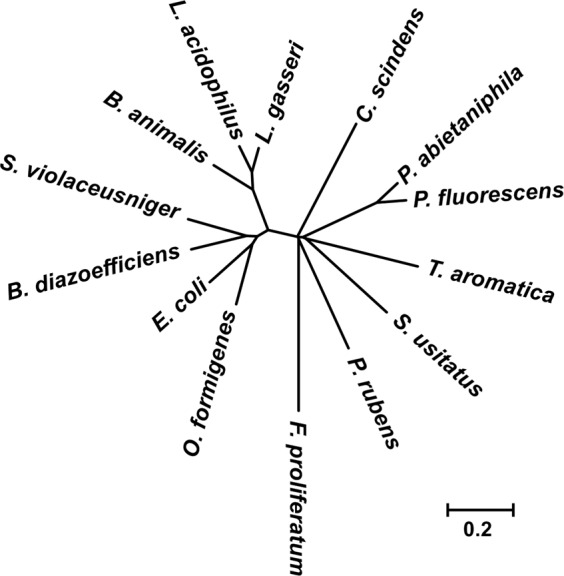
Unrooted phylogenetic tree of formyl-CoA transferase sequences. Proteins were aligned using CLUSTALX. Alignments were used for tree reconstruction. The organisms used were *Bifidobacterium animalis* (GenBank accession number WP004219152), *Bradyrhizobium diazoefficiens* USDA110 (NP769796), *Clostridium scindens* (AAC45415), *Escherichia coli* CFT073 (WP000106774), *Fusarium proliferatum* (AMB48876), *Lactobacillus acidophilus* NCK1728 (AAV42286), *Lactobacillus gasseri* ATCC 3323 (WP144231654), *Oxalobacter formigenes* Ox-B (AAC45298), *Penicillium rubens* Wisconsin 54–1255 (CAP92691), *Pseudomonas fluorescens* BBc6R8 (WP003209938), *Solibacter usitatus* Ellin 6076 (ABJ84069), *Streptomyces violaceusniger* Tu 4113 (AEM82356), and *Thauera aromatica* (AAF89841).

**Figure 4:**
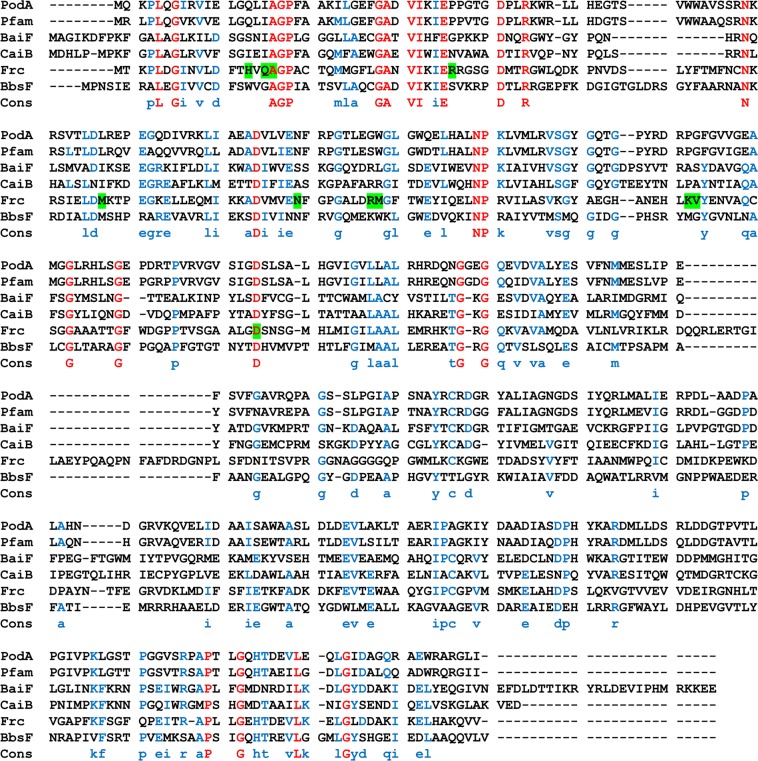
Sequence alignment of formyl-CoA transferases. Pfam02515, CoA transferase (WP003209938); Baif, putative cholate CoA transferase (AAC45415); CaiB, (*R*)-carnitine CoA transferase (CAA52112); Frc, formyl-CoA transferase (AAC45298); and BbsF, (*R*)-benylsuccinate CoA transferase (AAF89841). Identical residues are shown in red; residues suggested to be involved in catalysis are shown in blue. CoA-contacting residues proposed in Frc are marked green.

Using whole-genome information from GenBank (accession no. SDG16549), we analyzed a gene encoding an oxalate-degrading enzyme in *P. abietaniphila* ATCC 700689^17^. Translated amino acid sequences of PodA (42 kDa) showed 98% similarity with *P. abietaniphila* ATCC 700689 and 82% with *P. fluorescens* BBc6R8. Figure 3 shows the phylogenetic relationships of several putative formyl-CoA transferases from organisms whose protein sequences were available. As expected, the formyl-CoA transferases from *P. abietaniphila* and *P. fluorescens* clustered together. Sequence analysis showed that PodA, a formyl-CoA transferase, belongs to pfam02515, the CaiB-BaiF family of enzymes with diverse functions including fatty acid racemases, carnitine dehydratase (CaiB), bile acid inducible operon protein F (BaiF), benzylsuccinate CoA-transferase (BbsF), and anaerobic toluene catabolic protein in the presence of toluene. Identical residues, shown in red, are located in half of the N-termini of the proteins and partially in the C-termini (Fig. 4).

### PodA is required for the inhibition of *Botrytis* infection

**Figure 5:**
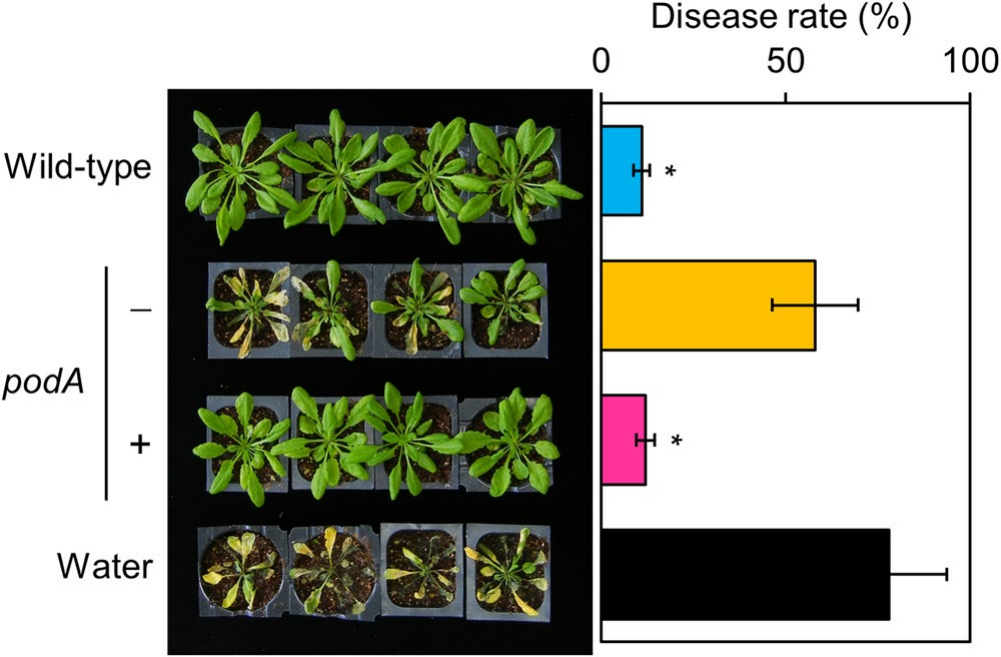
PodA is required for the inhibition of *Botrytis* infection. Four-week-old *Arabidopsis* plants were sprayed with suspensions (OD_600_ value 1.0) of the wild-type ODB36, *podA*^−^ mutant (−), and *podA* complementation (+) strains. After incubation for 2 days, a *B. cinerea* spore suspension (5 × 10^5^ spores/mL) was prepared using sterilised water. The spore suspension was sprayed until run-off occurred. Inoculated plants were placed in an opaque plastic box lined with saturated paper towels, the lids were removed after 2 days, and disease development was observed for 12 days and measured. Wild-type ODB36 strain exhibited significant disease suppression. The *podA*^−^ mutant did not inhibit *Botrytis* infection; the defect was recovered by *podA* complementation. Values are averages of duplicate assays; error bars represent the range. Asterisks denote significant differences from the water control (*p < 0.05; Student’s *t* test).

To investigate the effect of the wild-type ODB36, *podA*^−^ (*podA*–*lacZY*) mutant, and *podA* complementation strains on *Botrytis* infection, we performed disease suppression assays. Wild-type strain exhibited significant disease suppression. The *podA*^−^ mutant did not inhibit *Botrytis* infection; the defect was recovered by *podA* complementation (Fig. 5). These results indicated that *podA* is critical for the inhibition of *Botrytis* infection in *P. abietaniphila*.

### PodA is required for the inhibition of oxalate toxicity

**Figure 6:**
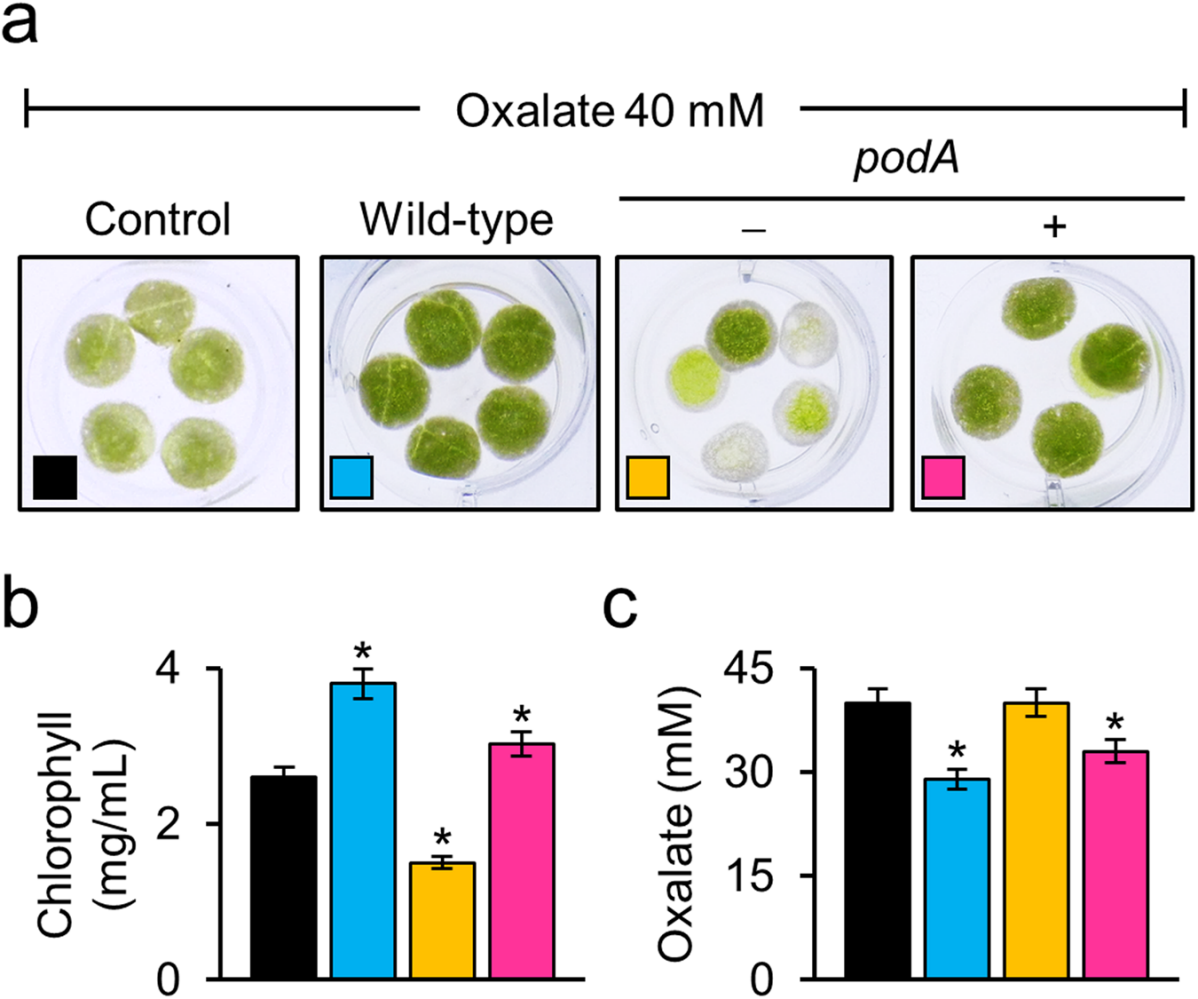
PodA is required for the inhibition of oxalate toxicity. (**a**) Tobacco leaf disc assays. Leaf discs were placed in suspensions of the wild-type, *podA*^−^ mutant (−), and *podA* complementation (+) strains supplemented with 40 mM sodium oxalate. After incubation for 5 days, oxalate toxicity was observed on the leaf discs in the *podA*^−^ mutant suspension but was reversed by *podA* complementation. (**b**) Chlorophyll in the leaf discs (**a**) was measured as described in the Methods section. (**c**) Remaining oxalate in suspensions (**a**) of the wild-type ODB36, *podA*^−^ mutant, and *podA* complementation strains. Values are averages of duplicate assays; error bars represent the range. Asterisks denote significant differences from the control (*p < 0.05; Student’s *t* test).

In tobacco leaf disc assays, oxalate toxicity, such as bleaching of leaf discs, was observed for the *podA*^−^ (*podA*–*lacZY*) mutant but was weak for the wild-type and *podA* complementation strains (Fig. 6a). This result was confirmed by the quantification of chlorophyll. Tobacco leaf discs treated with the *podA*^−^ mutant showed bleaching or a low chlorophyll level. These defects were restored to near wild-type levels by *podA* complementation (Fig. 6b). Also, oxalate degradation was induced by treatment of the wild-type and *podA* complementation strains (Fig. 6c).

### Overexpression, purification, and oxalate-degrading and formate conversion activities of PodA protein

**Figure 7:**
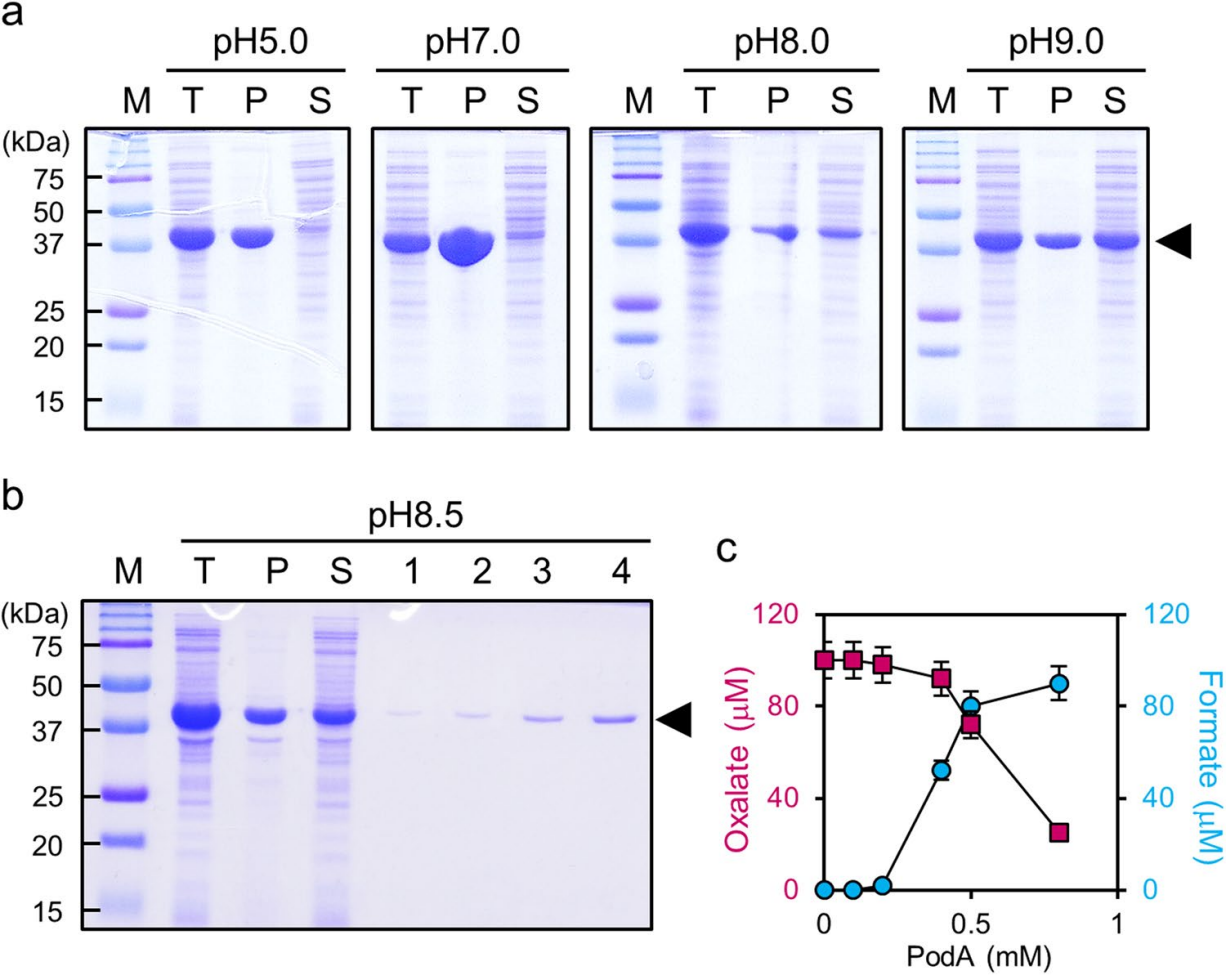
Overexpression, purification, and oxalate-degrading and formate conversion activities of PodA–His. (**a**) Sodium dodecyl sulphate polyacrylamide gel electrophoresis (SDS-PAGE) of PodA–His expression. The centrifuged cells of *E. coli* BL21(DE3) expressing PodA–His protein were subjected to sonic oscillation in cold 200 mM potassium phosphate buffer at pH 5.0–9.0. Each 10 µL sample was dissolved in 10 µL of 2 × loading buffer and boiled for 3 min at 100 °C. Final volumes of 10 µL were loaded in each lane. M, molecular mass standards; T, total cell extracts; P, pellet; S, supernatant of BL21(DE3) harbouring pLY205 after IPTG induction. (**b**) SDS-PAGE of PodA–His purification. Cell free sonic extract was worked up for purification using a Ni-NTA spin column. The purified protein eluted with 0.5 M imidazole was dialysed. Lanes 1–4: 1–, 2–, 5–, and 10–μL final elutions, respectively, containing PodA–His. Separation was conducted by 10% SDS-PAGE. Bands were visualised after staining with Coomassie Blue. The band corresponding to PodA–His is indicated. (**c**) Oxalate-degrading (closed square) and formate conversion (closed circle) activities of PodA–His determined using an oxalate colorimetric assay kit and a formate colorimetric assay kit, respectively. Values are averages of duplicate assays; error bars represent the range.

We overexpressed and purified *P. abietaniphila* ODB36 PodA from *Escherichia coli* BL21(DE3) carrying pLY205 (pET21b::*podA*). The *podA* gene encodes a protein of 396 amino acid residues with a calculated molecular weight of 42 kDa. To confirm that the *podA* gene encodes an oxalate-degrading enzyme, it was expressed under control of the T7 promoter in *E. coli*. Upon induction of *E. coli* BL21(DE3) harbouring pLY205 with IPTG, a His-tagged protein with a molecular mass of around 42 kDa was produced; approximately half of the PodA–His protein was expressed in soluble bodies at pH 8.0–9.0 (Fig. 7a). PodA–His was purified to near homogeneity using Ni-NTA affinity chromatography (Fig. 7b). The oxalate colorimetric assay kit revealed that PodA–His (0.8 mM) exhibited 75% oxalate-degrading activity (Fig. 7c). Using a formate colorimetric assay kit, we measured the formate conversion activity of PodA–His; the amount of degradation confirmed that oxalate was consistently converted to formate (Fig. 7c).

### Inhibition of oxalate toxicity by PodA–His protein

**Figure 8:**
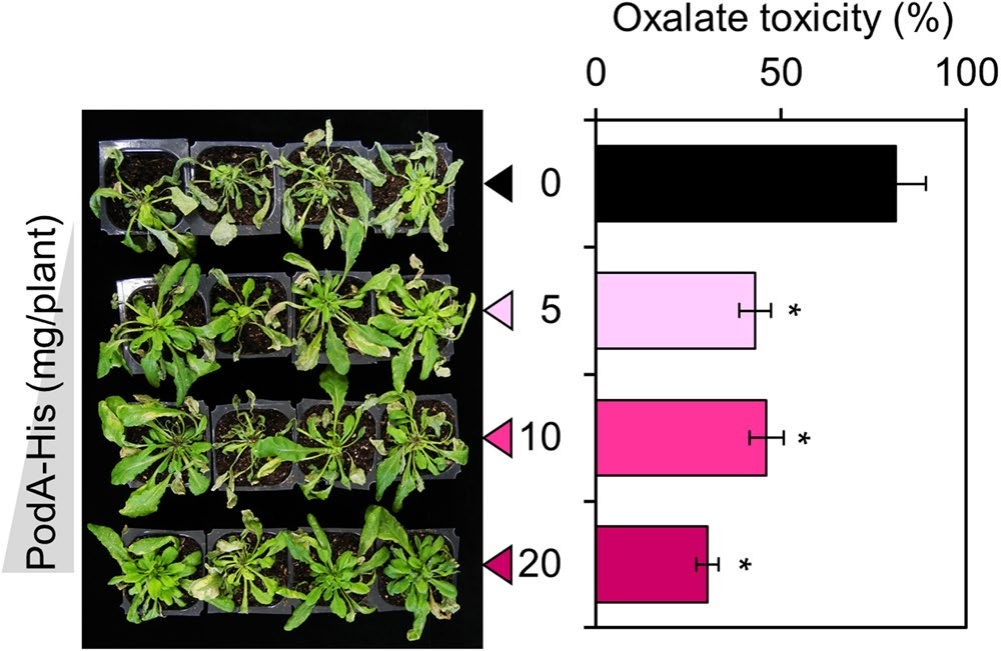
Inhibition of oxalate toxicity by PodA–His. Image captured 5 days after inoculation; oxalate toxicity rate (%). Six-week-old *Arabidopsis* plants were sprayed with PodA–His suspension. After incubation for 1 day, the plants were sprayed with an oxalate suspension (20 mM; 1 mL/plant). Oxalate toxicity was monitored for 5 days. PodA–His exhibited considerable and concentration-dependent inhibition of oxalate toxicity. Values are averages of duplicate assays; error bars represent the range. Asterisks denote significant differences from the control (*p < 0.05; Student’s *t* test).

To investigate the inhibitory effect of PodA–His protein on oxalate toxicity, we used a standard *A. thaliana*–oxalate (plant–toxin) system. Oxalate-sprayed plants exhibited an 81% oxalate toxicity rate, while PodA–His-sprayed plants showed significant and concentration-dependent inhibition of oxalate toxicity (Fig. 8).

## Discussion

Grey mould causes damage during the cultivation, storage, and transportation of fresh vegetables and soft-pulling fruits^1–3^. Efforts have been made to control grey mould using antifungal bacteria, but this is complicated by the stability of antimicrobial substances in fresh vegetables and the emergence of resistance^4,5^. For this reason, we investigated the possibility of suppressing grey mould using non-antifungal ODB, similar to the use of a non-antagonistic bacterium to control *Salmonella enterica*, which causes food poisoning^18^.

In this study, we isolated ODB from the surfaces of spinach and strawberries and evaluated their disease control activity. We also explored potential correlations between oxalate-degrading ability and disease inhibitory effects of the ODB isolates. Previous studies have used antagonistic ODB^13^ or ODB with unknown antagonistic effects^12^. Therefore, unlike these previous studies, we attempted to exclude antagonistic effects by excluding *P. fluorescens* ODB5, which exhibited antifungal activity; we selected bacterial strains *M. zatmanii* ODB35 and *P. abietaniphila* ODB36 for further testing of dose response to disease suppression. Significant suppression of *P. abietaniphila* ODB36 against grey mould is shown in Fig. 2. We expect that biological control of fungal pathogens can be achieved by neutralising pathogenic factors and inhibiting the occurrence of resistant individuals by applying a method that does not kill pathogens.

The disease suppression effect of ODB35, which has the highest oxalate-degrading ability, did not reach that of ODB36. This result is likely due to the slow growth rate of ODB35. As shown in Fig. 1, oxalate-degradation activity was calculated by multiplying the A_450_ value by bacterial growth. ODB35 exhibited relatively slow growth, resulting in a high value. These results suggest that the growth rate of ODB on the plant surface is important to its efficacy as a disease control agent. The aim of this study was to evaluate the oxalate-degrading ability of bacteria in disease control; we finally selected ODB36 as the optimal agent among those examined. Growth on plant surfaces was not investigated in this study.

Interestingly, the wild-type and *podA* complementation strains eliminated the toxicity of 40 mM oxalate, such as bleaching of tobacco leaf discs, but the *podA*^−^ mutant showed bleaching due to no oxalate toxicity. The remaining oxalate concentration in the wild-type and *podA*^−^ complementation strains was lower than that of the *podA*^−^ mutant strain. These data suggest that *podA* contributes to the degradation of oxalate. These results are in good agreement with the finding that grey mould control is by oxalate degradation in *P. abietaniphila*.

Family III CoA transferases include formyl-CoA:oxalate CoA transferase^16^, succinyl-CoA:(*R*)-benzylsuccinate CoA transferase^19^, (*E*)-cinnamoyl-CoA:(*R*)-phenyllactate CoA transferase^20^, and butyrobetainyl-CoA:(*R*)-carnitine CoA transferase^21^. These CoA transferases occur in prokaryotes and eukaryotes and catalyze CoA transfer reactions in a highly substrate- and stereo-specific manner^22^. Additionally, the PodA exhibited 81% identity (89% similarity) with Pfam02515, and 30% identity (47% similarity) with *O. formigenes* formyl-CoA transferase, which was the first member of family III of CoA transferases to be characterized^22^.

In this study, we did not quantify the formate concentration in ODB because *P. abietaniphila* strains contain several genes encoding formate dehydrogenase subunits, which catalyse the oxidation of formate to carbon dioxide. However, we performed protein overexpression and enzyme activity assays and confirmed that PodA–His converted oxalate to formate, which plays a role in the biosynthesis of many compounds in energetic metabolism and signal production related to stress in plants^23^.

**Figure 9:**
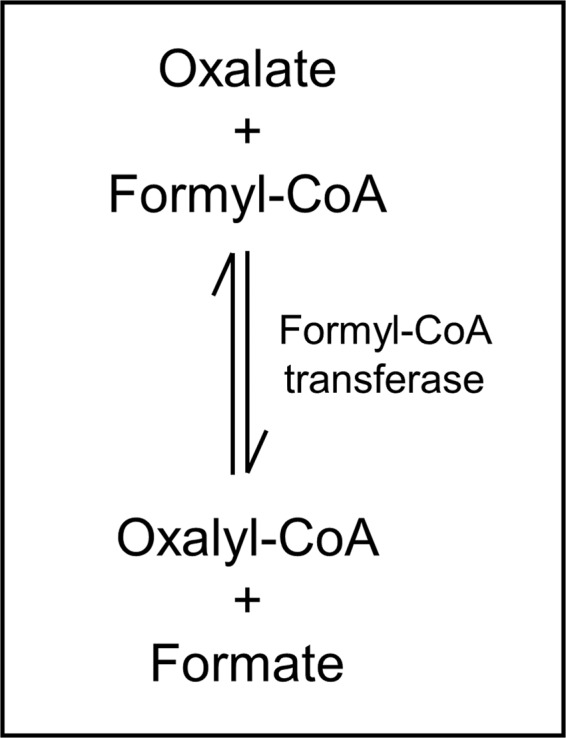
Oxalate degradation by formyl-CoA transferase. The two substrates, oxalate and formyl-CoA, are converted into the two products formate and oxalyl-CoA.

In plants, three types of oxalate-degrading enzymes are known, namely, oxidase (OXO), decarboxylase (OXDC), and acetylases, among which four enzymes (oxalyl-CoA synthetase [AAE3], oxalyl-CoA decarboxylase [OXDE], formyl-CoA hydrolase [FXH], and formyl-CoA dehydrogenase [FXDE]) act in stages^24–26^. In enzymatic analysis of oxalate degradation of bacteria, formyl-CoA transferase (EC 2.8.3.16) catalyzes the chemical reaction, which is demonstrated in Fig. 9^15,16^. The two substrates, oxalate and formyl-CoA, are converted into the two products oxalyl-CoA and formate. Formyl-CoA is required for the conversion of oxalate to oxalyl-CoA by formyl-CoA transferase^16^. However, the inhibitory effect of PodA on oxalate toxicity in *Arabidopsis* suggests the presence of formyl-CoA as a substrate. It is possibile that formyl-CoA derived from *Arabidopsis*-resident microorganisms acts as a substrate for PodA.

Previous studies have explored disease control in transgenic crops using plant-derived oxalate-degrading genetic resources. Barley oxalate oxidase transgenic peanuts showed enhanced resistance against *Sclerotinia minor*^27^. Soybean plants expressing wheat oxalate oxidase were resistant to *Sclerotinia sclerotiorum*^28,29^. These preliminary studies suggest that developed oxalate degradative genetic resources can be introduced into crops and inspire the development of transgenic plants with related abilities.

In conclusion, to overcome the competing processes of biological control and occurrence of resistant species, we investigated non-antimicrobial genetic resources for biological control of plant pathogens. Oxalate, a key pathogenic factor of necrotrophic fungi, was targeted to disarm, but not kill, plant pathogens. Oxalate-degrading bacteria were isolated and identified, and their activity was evaluated. Using disease suppression assays, *P. abietaniphila* ODB36 was finally selected for further investigation. PodA from ODB36, which encodes formyl-CoA transferase, was overexpressed and assayed using oxalate degradation tests. Bacterial cells of both ODB36 and enzyme PodA were found to be directly applicable in the field to control grey mould. The *podA* gene can also be applied for the development of transgenic resistant cultivars against grey mould.

## Methods

### Isolation of ODB

Surfaces of strawberries and spinach were swabbed with sterilised swabs, which were then streaked onto OM medium with 1.5% agar and incubated at 28 °C. After 7 days, single colonies were re-streaked onto OM agar medium for purification.

### Bacterial oxalate-degrading activity

Oxalate-degrading activity was evaluated using an oxalate colorimetric assay kit according to the supplier’s protocol. Optical density (OD_600_) was measured in bacterial cultures grown in OM broth plus 1/10 TSB at 28 °C for 3 days. After centrifugation, the supernatants were transferred into 96-well plates and mixed with 2 μL oxalate converter, which was supplied with the kit. The mixtures were incubated at 37 °C for 1 h, and 50 μL prepared reaction mix (46 μL buffer; 2 μL oxalate enzyme mix, and 2 μL oxalate probe; all reagents were supplied with the kit) was added to each well containing standard or samples. Then the plates were incubated at 37 °C for 1 h and absorbance was measured at 450 nm (A_450_). The values reporting the amount of oxalate remaining in the sample were normalized to culture growth by multiplying the A_450_ value for the non-degraded oxalate amount by the OD_600_ of the culture. The lower the value, the higher the degradation activity.

### 16S rRNA gene sequencing

To confirm the identities of the ODB, the 16S rRNA gene was amplified and sequenced using the primers 27mF (5′-AGAGTTTGATCMTGGCTCAG-3′) and 1429mR (5′-GGYTACCTTGTTACGACTT-3′). Total DNA was extracted using the Wizard Genomic DNA Purification Kit (Promega, Madison, WI, USA) following the manufacturer’s instructions. Polymerase chain reaction (PCR) was performed using a T100 thermal cycler (Bio-Rad, Hercules, CA, USA) with PCR polymerase (AccurPower PCR Premix; Bioneer, Daejeon, South Korea), 1 μL target DNA, and 1 mM each primer at 98 °C for 2 min, followed by 30 cycles of denaturation at 98 °C for 30 s, annealing at 55 °C for 30 s, and extension at 70 °C for 1 min, followed by a final extension at 72 °C for 4 min. The amplified products were separated by electrophoresis in 0.8% (w/v) agarose gels. PCR amplification yielded a single visible DNA product, whose band was cleaved from the ethidium bromide (EtBr)-stained gel and purified using a 200-p Expin Gel SV kit (GeneAll biotechnology, Seoul, Korea), following the manufacturer’s instructions. Purified PCR products were sequenced by Macrogen Services (Daejeon, Korea) in both directions using previously described primers^30^. DNA sequences were analyzed using the BLASTn program. DNA sequences of the 16S rRNA gene were compared with those in the National Center for Biotechnology Information (NCBI) GenBank database (http://www.ncbi.nlm.nih.gov/blast/).

### Antifungal activity test

Among the ODB, the antifungal activity of the three isolates (ODB5, ODB35, and ODB36) showing the highest oxalate-degrading activity was tested against *B. cinerea*, *A. alternata*, and *S. cerevisiae*. *Botrytis cinerea* and *A. alternata* spores were spread on half potato dextrose agar with half protease peptone (PDP) and 1.5% agar. *Saccharomyces cerevisiae* cells were embedded in PDP agar. The ODB suspension (10 μL) was dropped onto the plate, which was then incubated at 28 °C for 48 h and the magnitude of inhibition of fungal growth was monitored.

### Disease suppression assay

*Arabidopsis thaliana* Columbia (Col-0) was grown in a growth chamber at 20 °C under a 16-h photoperiod. Bacterial suspensions were prepared by resuspending the strains using sterilised water to adjust the OD_600_ values to the corresponding ranges (0.7–1.0). We sprayed 3-week-old *Arabidopsis* plants with the ODB suspensions (5 mL/plant). After 2 days of incubation, a *B. cinerea* spore suspension (5 × 10^5^ spores/mL) was prepared using sterilised water. The spore suspension was sprayed until run-off. Inoculated plants were placed in an opaque plastic box lined with saturated paper towels, the lids were removed after 2 days, and disease development was observed for 16 days and measured according to the following calculation: (no. diseased leaves/no. inoculated leaves) × 100.

### Sequence alignment and phylogenetic analysis

Protein sequences obtained from NCBI (https://www.ncbi.nlm.nih.gov/) were aligned and utilized to generate an unrooted phylogenetic tree using the neighbor-joining method (CLUSTALX software).

### Construction of the *podA*^−^ mutant and *podA* complementation strains

To generate the *podA*^−^ mutant, plasmid pLY201, carrying the internal fragment of the *podA* gene was constructed using ODB36 genomic DNA as the PCR template and PodAE (5′-AAGATACTGGGTGAGTTT-3′) and PodAK (5′-GGTACCCATCACCAGTTTCGGATT-3′) as primers. The amplified region (291 bp) was purified from an agarose gel and ligated into the pGEM-T Easy Vector System (Promega, Mannheim, Germany) to generate pLY201, which was confirmed by sequencing. pLY201 fragments generated by digestion with the restriction enzymes *EcoR*I and *Kpn*I (TaKaRa Bio Inc., Kusatsu, Japan) were purified after electrophoresis from an agarose gel and inserted into the suicide vector pVIK112^31^, generating pLY207. The resulting construct, pLY207, was transferred into *E. coli* S17-1 *λpir* and introduced into *P. abietaniphila* ODB36 by conjugation, generating *podA*–*lacZY*. The *lacZY* reporter gene fusion insertion mutants were selected based on the kanamycin-resistance phenotype and confirmed by PCR with primers that annealed upstream of the truncated fragments of *podA*, PodPro (5′-ATGCAAAAGCCTTTGCAAGGG-3′) and LacFuse (5′-GGGGATGTGCTGCAAGGCG-3′).

To construct the *podA* complementation strain, wild-type coding sequences with P*lac* were cloned into the broad-host vector pLAFR3. Coding sequences were amplified by PCR from ODB36 genomic DNA, using the primers PodAH (5′-GGCAAGCTTCTGAAACAGGAAACAGCTATGCAAAAGCCTTTGCAAGGG-3′) and PodAP (5′-GGCCTGCAGTCAAATCAATCCGCGAGCGCG-3′) and Phusion polymerase. Amplicons were ligated into pGEM-T Easy, confirmed by sequencing, excised by restriction enzyme cleavage, and ligated with appropriately cleaved pLAFR3. Plasmid pLY233 harbouring P*lac*–*podA* was verified by restriction digestion and sequencing prior to conjugation with *P. abietaniphilae* cells.

### Leaf disc assays and chlorophyll quantification

Tobacco leaf disc assays^32^ and chlorophyll quantification assays^33^ were performed as described previously.

### Overexpression and purification of PodA–His protein

We overexpressed PodA in *E. coli* by amplifying the coding region of *podA* using chromosomal DNA of ODB36 as a template with the primers PodN (5′-GGCCAT**ATG**CAAAAGCCTTTGCAAGGG-3′) with initiating ATG (bold) and PodX (5′-GGCCTCGAGAATCAATCCGCGAGCGCGCCA-3′) omitting stop codon, to which *Nde*I and *Xho*I sites (underline) were added. The amplified product was cloned at *Nde*I/*Xho*I site of pET21b containing a six-His tag at C-terminal (Novagen, Darmstadt, Germany) to generate pLY205. PodA–His was overexpressed in *E. coli* BL21(DE3), as described by the manufacturer (Novagen, Darmstadt, Germany). Cell free extracts were prepared by subjecting a suspension of 5 g centrifuged cells (wet weight) in 40 mL cold 0.2 M potassium phosphate or 0.2 M glycine-NaOH buffer (pH 5.0–9.0) to sonic oscillation in a solicitor (VCX130; Sonics & Materials Inc., Newtown, CT, USA) for 5 min at 4 °C. The sonic extract was centrifuged at 4 °C and 10,000 × *g* for 10 min. The supernatant solution was worked up for purification using a Ni-NTA spin column (Qiagen, Valencia, CA, USA). The eluted protein was dialyzed with the same buffer used for sonic oscillation, and the concentration of the purified protein was measured via the Bradford method with bovine serum albumin as the standard^34^.

### Oxalate degradation and formate conversion activities of PodA–His

The oxalate-degrading and formate conversion activities of purified PodA–His were evaluated using an oxalate colorimetric assay kit and a formate colorimetric assay kit, respectively, according to the supplier’s protocol (BioVision Inc., Milpitas, CA, USA).

### Inhibition of oxalate toxicity by PodA–His protein

*Arabidopsis thaliana* Columbia (Col-0) was grown in a growth chamber at 20 °C under a 16-h photoperiod. Protein suspensions were prepared by resuspending PodA–His in potassium phosphate buffer (pH 8.5) to 1, 2, and 4 mg/mL. We sprayed 6-week-old *Arabidopsis* plants with PodA–His protein suspensions (5 mL/plant). After incubation for1 day, the plants were sprayed with a sodium oxalate suspension (20 mM; 1–mL/plant). Oxalate toxicity was observed for 5 days and calculated according to the following equation: (no. of bleached leaves/total no. of leaves) × 100.

## Supplementary information

Supplementary Fig. S1.
