# Supplementary material for: Grey mould control by oxalate degradation using non-antifungal Pseudomonas abietaniphila strain ODB36
Source: Sci Rep. 2020 Jan 31;10:1605. doi: 10.1038/s41598-020-58609-z (PMC6994688; doi:10.1038/s41598-020-58609-z)
Supplement: Supplementary file 1 — Supplementary Fig. S1. [file 41598_2020_58609_MOESM1_ESM.docx]

**Supplementary Information**

**Grey mould control by oxalate degradation using non-antifungal *Pseudomonas abietaniphila* strain ODB36**

**Yeyeong Lee^1^, Okhee Choi^2^, Byeongsam Kang^3^, Juyoung Bae^3^, Seunghoe Kim^1^, Jinwoo Kim^1,2,3^***

^1^Department of Plant Medicine, Gyeongsang National University, Jinju 52828, Republic of Korea; ^2^Institute of Agriculture & Life Science, Gyeongsang National University, Jinju 52828, Republic of Korea; ^3^Division of Applied Life Science, Gyeongsang National University, Jinju 52828, Republic of Korea.

*Corresponding author: Jinwoo Kim, Institute of Agriculture & Life Science, Gyeongsang National University, Jinju 52828, Republic of Korea; Tel: +82-55-772-1927; Fax: +82-55-772-1929; Email: jinwoo@gnu.ac.kr

**Figure S1.**

**
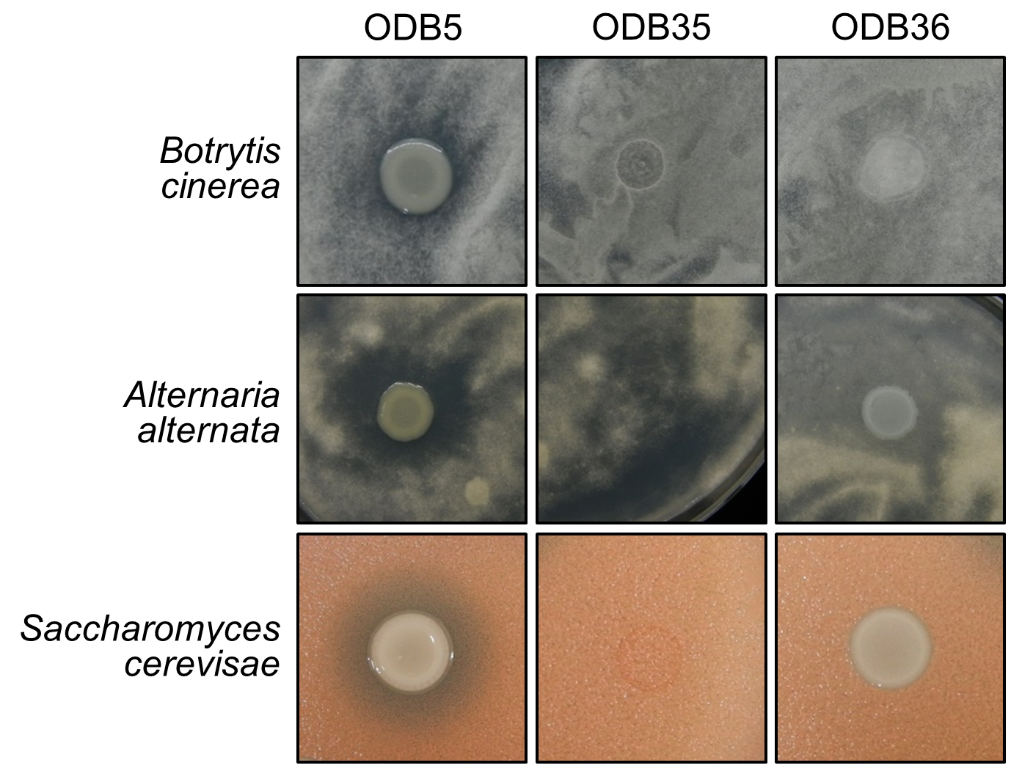
**

**Figure S1. Anti-fungal activity of oxalate-degrading bacterial isolates.** *Botrytis cinerea* and *A. alternata* spores were spread on half potato dextrose agar with half protease peptone (PDP) and 1.5% agar. *Saccharomyces cerevisiae* cells were embedded in PDP agar. The ODB suspension (10 μL) was dropped onto the plate, which was then incubated at 28°C for 48 h.
